# Apnoeic oxygenation for emergency anaesthesia of pre-hospital trauma patients

**DOI:** 10.1186/s13049-020-00817-7

**Published:** 2021-01-07

**Authors:** Kate Crewdson, Ainsley Heywoth, Marius Rehn, Samy Sadek, David Lockey

**Affiliations:** 1 London’s Air Ambulance, London, UK; 2 Intensive Care Unit, Gate 37, Level 2, Brunel Building, Southmead Hospital, Southmead Road, Bristol, BS10 5NB UK; 3 Essex & Herts Air Ambulance Trust, Essex, UK; 4 Department of Research, Norwegian Air Ambulance Foundation, Oslo, Norway; 5 Faculty of Health Sciences, University of Stavanger, Stavanger, Norway; 6 Blizard Institute, Queen Mary University of London, London, UK

**Keywords:** Apnoeic oxygenation, Pre-oxygenation, Intubation, Trauma, Emergency

## Abstract

**Background:**

Efficient and timely airway management is universally recognised as a priority for major trauma patients, a proportion of whom require emergency intubation in the pre-hospital setting. Adverse events occur more commonly in emergency airway management, and hypoxia is relatively frequent. The aim of this study was to establish whether passive apnoeic oxygenation was effective in reducing the incidence of desaturation during pre-hospital emergency anaesthesia.

**Methods:**

A prospective before-after study was performed to compare patients receiving standard care and those receiving additional oxygen via nasal prongs. The primary endpoint was median oxygen saturation in the peri-rapid sequence induction period, (2 minutes pre-intubation to 2 minutes post-intubation) for all patients. Secondary endpoints included the incidence of hypoxia in predetermined subgroups.

**Results:**

Of 725 patients included; 188 patients received standard treatment and 537 received the intervention. The overall incidence of hypoxia (first recorded SpO_2_ < 90%) was 16.7%; 10.9% had SpO_2_ < 85%. 98/725 patients (13.5%) were hypoxic post-intubation (final SpO_2_ < 90% 10 minutes post-intubation).

Median SpO_2_ was 100% vs. 99% for the standard vs. intervention group. There was a statistically significant benefit from apnoeic oxygenation in reducing the frequency of peri-intubation hypoxia (SpO_2_ < =90%) for patients with initial SpO_2_ > 95%, *p* = 0.0001. The other significant benefit was observed in the recovery phase for patients with severe hypoxia prior to intubation.

**Conclusion:**

Apnoeic oxygenation did not influence peri-intubation oxygen saturations, but it did reduce the frequency and duration of hypoxia in the post-intubation period. Given that apnoeic oxygenation is a simple low-cost intervention with a low complication rate, and that hypoxia can be detrimental to outcome, application of nasal cannulas during the drug-induced phase of emergency intubation may benefit a subset of patients undergoing emergency anaesthesia.

## Background

Rapid and effective airway management is a priority for major trauma patients. Advanced airway interventions are necessary for a small subgroup of severely injured patients [1], in whom basic airway interventions are inadequate to maintain oxygenation and ventilation. Pre-hospital emergency anaesthesia (PHEA) is required to facilitate tracheal intubation and ventilation.

Adverse events associated with intubation occur more frequently during emergency airway management particularly when repeated attempts at laryngoscopy are required [2–4]. Hypoxia is one of the more commonly occurring adverse events and has been demonstrated to occur in 9.2% of patients during the first attempt at intubation in an emergency setting, increasing to 37.8% of patients where there are repeated intubation attempts [4]. Pre-hospital data suggests that between 10.9 and 18.3% of patients undergoing PHEA experience episodes of hypoxaemia [5–7], and these episodes are associated with an increase in morbidity and mortality [8].

Preoxygenation is a universally accepted method of reducing hypoxia during the drug-induced apnoeic phase of induction of anaesthesia. Achieving peripheral oxygen saturations above 93% extends the time taken before the onset of hypoxia during induction. At lower values, the dissociation of oxygen from haemoglobin takes place on the steep portion of the oxyhaemoglobin dissociation curve and oxygen saturations drop rapidly [9]. Standard practice for pre-oxygenation typically involves use of a reservoir bag supplying a high concentration of inspired oxygen. The benefit of additional apnoeic oxygenation has been widely debated but has been reported to provide benefit in terms of increased peri-intubation oxygen saturation, decreased rates of hypoxia, and increased first-pass intubation success without hypoxia [10]. Nasal oxygenation using low-flow nasal prongs is a recognised low-risk and easily administered procedure for providing passive apnoeic oxygenation in the pre-intubation and peri-intubation phases of emergency anaesthesia. This intervention has previously been reported to reduce desaturation rates by 6% [6].

This study aims to investigate whether the introduction of apnoeic oxygenation would reduce the frequency of desaturation in a population of trauma patients undergoing PHEA. The null hypothesis states that there is no difference in median oxygen saturations observed between the two study groups (before and after intervention) in the peri-RSI period, defined as 2 minutes pre-intubation to 2 minutes post-intubation.

## Methods

### Study setting

A prospective before-after study was performed which included all trauma patients undergoing PHEA performed by two physician-paramedic manned pre-hospital services. The services together attend over 3000 patients per year. Doctor–paramedic teams are delivered by helicopter and fast response cars. Flight paramedics in the ambulance control room dispatch the services and specific dispatch criteria target critically ill or injured patients. A ground ambulance is always dispatched in addition to the physician–paramedic team. Trauma patients attended by the doctor-paramedic teams of the two UK pre-hospital services who underwent PHEA were included in the study. Exclusion criteria were patients intubated prior to the arrival of the physician-paramedic team, in cardiac arrest on arrival of the pre-hospital physician-paramedic team, with nasal haemostatic devices in situ for maxillofacial haemorrhage, who had a medical event (non-traumatic cardiac arrest or cerebrovascular event) immediately preceding their traumatic episode and less than 16 years of age.

The delivery of PHEA within the pre-hospital trauma services in which the study was based is standardised and delivered to standard operating procedures. The decision to anaesthetise the study patients was made at the attending clinician’s discretion based on defined indications. All patients were pre-oxygenated for 2 minutes prior to intubation – this was usually performed with a standard non-rebreathing mask, but if the patient had SpO_2_ of 90% or lower, gentle bag-valve-mask ventilation was applied. All patients undergoing PHEA were fully monitored and anaesthetised using ketamine as an induction agent, with or without the addition of the synthetic opioid, fentanyl. A muscle relaxant, rocuronium, was also administered. The drug doses were based on the physiological status and estimated weight of the patient; opioids were reduced or omitted if the patient is considered to have severe cardiovascular compromise. Patients were fully monitored using standard monitoring meeting the Association of Anaesthetists of Great Britain and Ireland recommendations [11]. Patients in the study were divided into two study groups, pre and post introduction of apnoeic oxygenation:

- ▪ Standard group: Patients received supplementary oxygen via a reservoir bag attached to an oxygen supply running at 15 L/min is applied to the patient prior to the start of PHEA.
- ▪ Intervention group: Patients received additional oxygen via nasal prongs at a flow rate of 15 L/min. The nasal prongs were applied when the decision to perform PHEA was made. The standard reservoir bag was applied to the patient as per normal practice and the nasal prongs remained in situ for the duration of PHEA.

The patients were sub-grouped as below according to the pre-induction SpO2 value (taken at 0 min) prior to data analysis.

- No hypoxia: SpO2 > 95%
- Mild / moderate hypoxia: SpO_2_ 85–95%
- Severe hypoxia: SpO_2_ < 85%

Apnoeic oxygenation was introduced into routine clinical practice within the service in February 2016. A training package was provided for all pre-hospital personnel prior to introduction of the intervention. This package included a review of current literature related to apnoeic oxygenation, a brief overview of the study, a questionnaire about the technique and a training moulage using apnoeic oxygenation. Apnoeic oxygenation was added to the mandatory PHEA challenge-respond checklist, which is routinely performed before induction of anaesthesia, to ensure use of the intervention in the post intervention phase. Data collection for the post-intervention phase was started after a two-week adjustment or ‘washout’ period to ensure apnoeic oxygenation had become embedded into routine practice.

Data for the trial was downloaded after arrival in hospital from the monitors used to record the pre-hospital patients’ vital signs after arrival in hospital. Both services used the Zoll X Series (Zoll, Boston, Massachusetts United States) monitor to record physiological data. Monitoring was commenced on arrival of the doctor-paramedic pre-hospital advanced care team and disconnected when the patient arrived at hospital. Data were continuously recorded and displayed as a waveform and numerical value which is updated every 30 s. SpO_2_ values were recorded for each patient from 2 minutes prior to the start of PHEA (during the pre-oxygenation period), until 10 minutes after PHEA had been performed.

The primary study outcome was the difference in median oxygen saturations observed between the two groups in the peri-RSI period, defined as 2 minutes pre-intubation to 2 minutes post-intubation for all patients and for patient subgroups. The null hypothesis was that there is no difference between the two groups.

Secondary outcomes included:

- The incidence of hypoxia, (defined as SpO_2_ values of 90% or less) in the peri-intubation period (2 min before and 2 min after intubation for all patients and for the defined patient subgroups.
- The incidence of severe hypoxia, defined as SpO_2_ values of 85% or less, in the peri-intubation period (2 min before and 2 min after intubation) for all patients and for patient subgroups.
- The incidence of hypoxia, defined as SpO_2_ values of 90% or less, in the post-intubation phase from intubation until 10 minutes post-intubation for all patients and for patient subgroups.
- The incidence of hypoxia, defined as SpO_2_ values of 90% or less, in the recovery phase from 2 minutes post intubation until 10 minutes post intubation for all patients and for patient subgroups.

Hypoxia rates were compared between groups using chi-squared tests. Differences between groups in SpO_2_ as a continuous variable were investigated using the Mann-Whitney U test.

The study was registered with Barts and the London NHS Trust as a Clinical Effectiveness study (no. 5856). No additional interventions were conducted or data recorded on patients studied. Ethical approval was therefore not required.

## Results

In total, 725 patients were included in the study; 188 patients were included in the standard treatment group and 537 in the intervention group. The overall incidence of hypoxia prior to pre-oxygenation and intubation (defined as initial SpO_2_ reading < 90%) was 16.7% (121 of 725 patients); 79 patients (10.9%) had SpO_2_ less than 85%. 98 of 725 patients (13.5%) were hypoxic post-intubation (defined as final SpO_2_ reading < 90%, observed at 10 minutes post intubation); 70 of these 98 patients (9.7%) were in the group of 79 patients who were initially hypoxic with SpO_2_ less than 85% prior to intubation (i.e. the group who could not be pre-oxygenated).

Median SpO_2_ in the standard group was 100% (95% CI (99,100), the median SpO_2_ in the intervention group was 99% (95% CI (98,100), *p* = 0.0001. The incidence of hypoxia, defined as SpO_2_ values of 90% or less, in the 2 min before and 2 min after intubation was 106/653, 16.2% in the standard group and 500/3181, 15.7% in the intervention group, *p* = 0.74 The incidence of hypoxia, in the period from intubation until 10 minutes post intubation was 177/1461, 12.1% in the standard group and 959/8037, 11.9% in the intervention group, *p* = 0.84. The results from the subgroup analysis are reported in Table 1 below.

**Table 1:** Subgroup analysis for study groups

Peri intubation phase 2 min pre to 2 min post intubation		Standard	Intervention	***P*** value
Median SaO_2_ (IQR)	All patients	100 (96,100)	99 (96,100)	*p* = 0.0001
	No hypoxia	100 (98,100)	100 (98,100)	*p* = 1.00
	Mild Moderate hypoxia	94 (83,97.75)	94 (88,99)	*p* = 1.00
	Severe hypoxia	79 (66.5,86)	83 (77,95)	*p* = 0.28
Total post intubation values / total 90 or less (% difference)	All patients	106/653 (16.2)	560/3659 (15.3)	0.546
	No hypoxia	24/380 (6.3)	54/1492 (3.6)	0.02
	Mild Moderate hypoxia	28/72 (38.9)	131/386 (33.9)	0.947
	Severe hypoxia	41/51 (80.4)	118/177 (66.7)	0.195
Total post intubation values / total 85 or less (% difference)	All patients	65/653 (10.0)	340/3659 (9.3)	0.864
	No hypoxia	7/380 (1.8)	20/1492 (1.3)	0.466
	Mild Moderate hypoxia	11/71 (15.3)	61/386 (15.8)	0.947
	Severe hypoxia	34/51 (66.7)	100/177 (56.5)	0.195
**Post intubation phase (intubation - 10 min)**	All patients	177/1464 (12.1)	959/8037 (11.9)	0.864
Total post intubation values / total 90 or less (% difference)	No hypoxia	43/1126 (3.8)	254/5928 (4.3)	0.476
	Mild Moderate hypoxia	39/164 (23.8)	377/1424 (26.5)	0.458
	Severe hypoxia	93/160 (58.1)	384/759 (50.6)	0.084
**Recovery phase (2 min post intubation to 10 min post intubstion**	All patients	139/1221 (11.4)	728/6396 (11.4)	0.998
Total post intubation values / total 90 or less (% difference)	No hypoxia	33/941 (3.5)	204/5121 (4.0)	0.488
	Mild Moderate hypoxia	31/138 (22.5)	280/1128 (24.2)	0.544
	Severe hypoxia	73/133 (54.9)	234/746 (31.4)	3.06E-07
Median SpO_2_ (IQR)	All patients	100 (96,100)	99 (96,100)	*p* = 0.0001
	No hypoxia	100 (98,100)	100 (98,100)	*p* = 1.00
	Mild Moderate hypoxia	94 (83,97.75)	94 (88,99)	*p* = 1.00
	Severe hypoxia	79 (66.5,86)	83 (77,95)	*p* = 0.28
Total 90 or less / total peri intubation values (% difference)	All patients	106/653 (16.2)	560/3659 (15.3)	0.546
	No hypoxia	24/380 (6.3)	54/1492 (3.6)	0.02
	Mild Moderate hypoxia	28/72 (38.9)	131/386 (33.9)	0.947
	Severe hypoxia	41/51 (80.4)	118/177 (66.7)	0.195
Total 85 or less / total peri intubation values (% difference)	All patients	65/653 (10.0)	340/3659 (9.3)	0.864
	No hypoxia	7/380 (1.8)	20/1492 (1.3)	0.466
	Mild Moderate hypoxia	11/71 (15.3)	61/386 (15.8)	0.947
	Severe hypoxia	34/51 (66.7)	100/177 (56.5)	0.195
**Post intubation phase (intubation - 10 min)**	All patients	177/1464 (12.1)	959/8037 (11.9)	0.864
Total 90 or less / total post intubation values (% difference)	No hypoxia	43/1126 (3.8)	254/5928 (4.3)	0.476
	Mild Moderate hypoxia	39/164 (23.8)	377/1424 (26.5)	0.458
	Severe hypoxia	93/160 (58.1)	384/759 (50.6)	0.084
**Recovery phase (2 min post intubation to 10 min post intubation)**	All patients	139/1221 (11.4)	728/6396 (11.4)	0.998
Total 90 or less / total post intubation values (% difference)	No hypoxia	33/941 (3.5)	204/5121 (4.0)	0.488
	Mild Moderate hypoxia	31/138 (22.5)	280/1128 (24.2)	0.544
	Severe hypoxia	73/133 (54.9)	234/746 (31.4)	*p* < 0.0001

Table 1 shows the 2 study groups (standard and intervention) subclassified by the degree of hypoxia was subclassified according to the pre-induction SpO2 value (taken at 0 min) as:

• No hypoxia: SpO2 > 95%

• Mild / moderate hypoxia: SpO_2_ 85–95%

• Severe hypoxia: SpO_2_ < 85%

The median SpO_2_ is the median value of all values recorded for the all patients during the peri-intubation phase

The categories reporting ‘total 90 or less’ and ‘total 85 or less’ refer to the number of patients who desaturated in the peri-intubation, post intubation, or recovery phase to an SpO_2_ or 90% or less, or 85% or less. This figure is reported as a proportion of the number of SpO_2_ values recorded for all patients in this group and subclassification

## Discussion

This is the first reported prospective study of the use of apnoeic oxygenation during PHEA in trauma patients. Hypoxia is a relatively common problem in patients undergoing emergency intubation; 16.7% of patients in this study were hypoxic prior to induction of anaesthesia. This is comparable to previous studies which report that up to 18% of trauma patients are hypoxic prior to airway intervention [7, 12, 13]. A single episode of hypoxia may worsen patient outcome, particularly in traumatic brain injury [8, 14, 15]. Reducing the time spent with low oxygen saturations may translate into an improvement in morbidity and mortality. Patients who have a SpO_2_ less than 93% prior to intubation, and multiple or prolonged intubation attempts are much more likely to desaturate [3]. A reduction in hypoxic episodes may be achieved using simple reproducible techniques such as apnoeic oxygenation. The physiology of apnoeic oxygenation is well-described; in the presence of a patent airway, there is a difference between the uptake of oxygen and the excretion of carbon dioxide at an alveolar level [16]. This discrepancy creates a negative pressure gradient that promotes the movement of oxygen into the lungs. Alveoli will continue to take up oxygen even without diaphragmatic movements or lung expansion. The presence of a continuous infusion of oxygen to the upper airways during drug-induced apnoea can supplement oxygenation levels [17].

Despite multiple previous studies, the clinical benefit of apnoeic oxygenation remains unproven. A positive association between apnoeic oxygenation and lower rates of hypoxaemia during emergency intubation has been demonstrated [10, 13, 18–20] but these findings are not universally reproducible. The application and delivery of apnoeic oxygenation varies between the studies, with some using 15 L/min via nasal cannulae and some studies using high flow oxygen at 50–60 L/min; endpoints also vary between studies. Results from two randomised controlled trials performed in the emergency setting could not demonstrate a benefit of apnoeic oxygenation [21, 22]. Studies conducted using pre-hospital data are retrospective; two studies have demonstrated a reduction in the incidence of hypoxia, but this has not reached statistical significance [6, 23]. This study provided apnoeic oxygenation at a rate of 15 L/min using nasal prongs but did not show a significant overall benefit in reducing the frequency of episodes of hypoxia during the drug-induced apnoeic phase of intubation. Apnoeic oxygenation may be beneficial where intubation is difficult and time to ventilation is prolonged. It can increase time to desaturation which reduces the incidence of desaturation and the frequency of hypoxic episodes in difficult or prolonged intubations [24–26].

Overall, the results of this study did not demonstrate a significant reduction in the incidence of hypoxia using apnoeic oxygenation during PHEA. It is likely that where pre-oxygenation has been adequately performed and the intubation is uncomplicated, minimal benefit will be observed with the addition of apnoeic oxygenation [24]. The intubation success rate for the services in which the study was conducted has previously been demonstrated to be 99.1 and 99.3% [27] with a first pass success rate of 93 and 87.5% [28]. There was a statistically significant difference in the median peri-intubation oxygen saturations between the two study groups in favour of the standard group, but this does not translate into any clinically relevant difference as neither result demonstrates clinically significant hypoxia.

Subgroup analysis was performed to assess the effect of apnoeic oxygenation on different categories of pre-intubation hypoxia. There was a statistically significant benefit from apnoeic oxygenation in the frequency of peri-intubation hypoxia (SpO_2_ of 90% or lower) for patients who started with normal oxygen saturations (> 95%) as shown in Table 1. The other significant benefit was observed in the recovery phase for the group of patients who were severely hypoxic prior to induction of anaesthesia.

Apnoeic oxygenation is a simple low-cost intervention with a low complication rate. The intervention currently lacks a firm evidence base but may be helpful in a difficult intubation or where intubation takes longer than usual. The ongoing controversy surrounding use of this technique does not necessarily mean that apnoeic oxygenation is ineffective in reducing the incidence of hypoxaemia associated with emergency intubation. This study used a pragmatic approach to try and investigate the effect of apnoeic oxygenation in a cohort of severely injured patients undergoing pre-hospital intubation. Although apnoeic oxygenation is unlikely to make a difference to the majority of trauma patients who are straightforward to oxygenate and easy to intubate, it has the potential to reduce the frequency, incidence, and duration of hypoxia in the minority of patients who are already hypoxic or require a longer time to intubate. Difficult pre-oxygenation is a risk factor for subsequent hypoxia, and up to 30% of patients can remain hypoxic, defined as SpO_2_ < 90%, after 5 min of pre-oxygenation [29], so apnoeic oxygenation is potentially very beneficial in the group of patients most vulnerable to hypoxia. A recent study comparing bag-mask ventilation to no ventilation during intubation of critically ill patients reported higher oxygen saturations and a lower incidence of severe hypoxemia in the bag-mask ventilation group [30]. This positive finding may be relevant to this study population because an alternative strategy to providing supplemental oxygenation in the apnoeic phase of induction might be to avoid the apnoeic phase altogether with bag-valve-mask ventilation.

### Limitations

This is a prospective-interventional study assessing whether apnoeic oxygenation is a beneficial intervention in PHEA. Whilst before-after studies are considered to be weaker methodology; at the time the study was designed there was relatively little data published on apnoeic oxygenation; the published data seemed to suggest a benefit and there was insufficient equipoise to conduct a randomised controlled study. Data for this study were collected using monitor downloads, introducing the possibility of drift and calibration errors. The manufacturers state the monitors potentially have up to 0.3% measurement error. It is not possible to collect specific information including patient demographics, injuries sustained or the indication for intubation. In addition, it was not possible to collect long-term patient outcome data such as survival to hospital discharge, functional outcome at discharge. The study is designed pragmatically to enable the research to be conducted in critically ill and injured patients. The clinical treatment provided is protocol-led and all clinicians working within the services are fully conversant with these guidelines. There may be occasional circumstances where care delivered deviates from the protocol – this is at the discretion of the attending clinician. The patient population used in this study – all trauma patients undergoing RSI, may not be representative of the patient group most likely to benefit from apnoeic oxygenation. However a study only including, for example, patients difficult to intubate or at most risk of desaturation would take a very large patient population or length of time to investigate. It is possible this study was underpowered to detect a significant difference in both statistical and clinical terms.

## Conclusions

In this study apnoeic oxygenation did not influence peri-intubation oxygen saturations but it did reduce the frequency and duration of hypoxia in the post-intubation period in specific patient groups. Given that apnoeic oxygenation is usually a safe and simple technique and that hypoxia can be detrimental to outcome, application of nasal cannulas during the drug-induced phase of emergency intubation may benefit a small subset of patients undergoing emergency intubation.

## Data Availability

Data sharing is not applicable to this article as no datasets were generated or analysed during the current study.
